# Spatial Pattern Detection of Tuberculosis: A Case Study of Si Sa Ket Province, Thailand

**DOI:** 10.3390/ijerph121215040

**Published:** 2015-12-17

**Authors:** Siriwan Hassarangsee, Nitin Kumar Tripathi, Marc Souris

**Affiliations:** 1 Remote Sensing and Geographic Information System Field of Study, School of Engineering and Technology, Asian Institute of Technology, P.O. Box 4, Klong Luang, Pathumthani 12120, Thailand; nitinkt@ait.asia (N.K.T.); Marc.Souris@ird.fr (M.S.); 2 UMR_D 190 “Emergence des Pathologies Virales”, IRD, Aix-Marseille University, EHESP, INSERM, IFS, 13385 Marseille, France

**Keywords:** tuberculosis, spatial autocorrelation, spatial scan statistic, Si Sa Ket, Thailand

## Abstract

This retrospective population-based study was conducted to analyze spatial patterns of tuberculosis (TB) incidence in Si Sa Ket province, Thailand. TB notification data from 2004 to 2008 collected from TB clinics throughout the province was used along with population data to reveal a descriptive epidemiology of TB incidences. Global clustering patterns of the occurrence were assessed by using global spatial autocorrelation techniques. Additionally, local spatial pattern detection was performed by using local spatial autocorrelation and spatial scan statistic methods. The findings indicated clusters of the disease occurred in the study area. More specifically, significantly high-rate clusters were mostly detected in Mueang Si Sa Ket and Khukhan districts, which are located in the northwestern part of the province, while significantly low-rate clusters were persistent in Kantharalak and Benchalak districts, which are located at the southeastern area.

## 1. Introduction

Tuberculosis (TB) is a respiratory infectious disease caused by the bacillus, *Mycobacterium tuberculosis*, which has been a global public health concern for decades. In April 1993, the World Health Organization (WHO) declared TB a global emergency [1]. According to the WHO, 22 countries, including Thailand, were considered high burden countries (HBCs) due to their large number of reported prevalence. In 2013, the WHO reported that HBCs accounted for more than 80% of the global notified cases [2]. 

In Thailand, the Ministry of Public Health (MOPH) reported that around 30%–40% of the Thai population was infected by the TB bacilli. This disease was placed among the top ten leading diseases under the National Disease Surveillance System. In 2010, the morbidity rate of the disease was 63.72 per 100,000 population. At the provincial level, Si Sa Ket, was ranked as the highest burden province for over a decade. The provincial morbidity rates were more than two folds compared to that of the national-wide rate. From 2008 to 2010, Si Sa Ket had morbidity rates of 164.33, 168.37, and 209.33 per 100,000 population respectively [3].

The government of Thailand and its partners have taken major steps to prevent, diagnose, and treat TB. Several studies have been conducted at the national, regional, provincial, district, and sub-district levels to investigate the epidemiology of this disease in Thailand. Although they claimed to reveal supportive information for TB control programs, spatial context such as distribution and clustering patterns of the disease, especially at finer level, have rarely been taken into account. 

In recent years, Geographical Information System (GIS) with spatial statistics—including spatial filtering and cluster analysis—has been applied to analyze and visualize the spatial patterns of TB. GIS and spatial scan statistics were used to detect the geo-spatial hotspots of TB in Almora district of India and found significant high-rate spatial and space-time clusters in three areas of the district [4]. The analysis tools were also used to investigate TB patterns in Dehradun city of India. Significantly high-rate clusters were detected in the area [5]. Couceiro and colleagues studied the pulmonary TB (PTB) incidence in Portugal by using GIS and spatial scan statistics incorporated with multivariate regression analysis and found that some areas were at higher risk of PTB than others because of high incidence of HIV/AIDS and sub-standard accommodation [6]. In Japan, space-time scan statistics were used to investigate TB clusters in Fukuoka [7]. Spatial clustering of TB was also detected in Madagascar. It was associated with socio-economic and patient care factors in Antananarivo City [8]. With respect to spatiotemporal clustering, three high incidence space-time clusters were identified in Portugal between 2000 and 2004 [9]. In South Africa, GIS and spatial analysis were utilized to study transmission patterns of the disease in a high-incidence area [10]. In an urban West Africa, spatial scan statistics were used to assess purely spatial and space-time clusters of TB in Greater Banjul [11]. Another study in Brazil revealed spatial patterns of the incidence of this disease and its relationship with socio-economic status [12]. In Beijing, GIS and spatial analysis were used to determine the role of migration in the transmission of the disease [13]. These examples would be evidences of GIS applications to define spatial pattern of TB or to identify its clusters.

Therefore, this study proposed to make use of GIS and spatial analysis in order to investigate spatial patterns of TB at the local authority organization (LAO) level of Si Sa Ket province in Thailand from 2004 to 2008. It aimed to characterize the epidemiology of TB in the province, evaluate whether observed geographic variations of TB incidence were random or statistically significant, identify the location of such phenomenon, and determine whether statistically significant excesses or deficits were temporary or time persistent. The findings would serve as a support tool for decision makers, academics, researchers, and practitioners to formulate more sound and targeted strategies for TB prevention and control.

## 2. Materials and Methods 

The method of this study was a retrospective investigation of reported tuberculosis cases collected from TB clinics in Si Sa Ket province between 2004 and 2008. The unit of analysis in this study is the LAO level.

### 2.1. Study Area 

**Figure 1:**
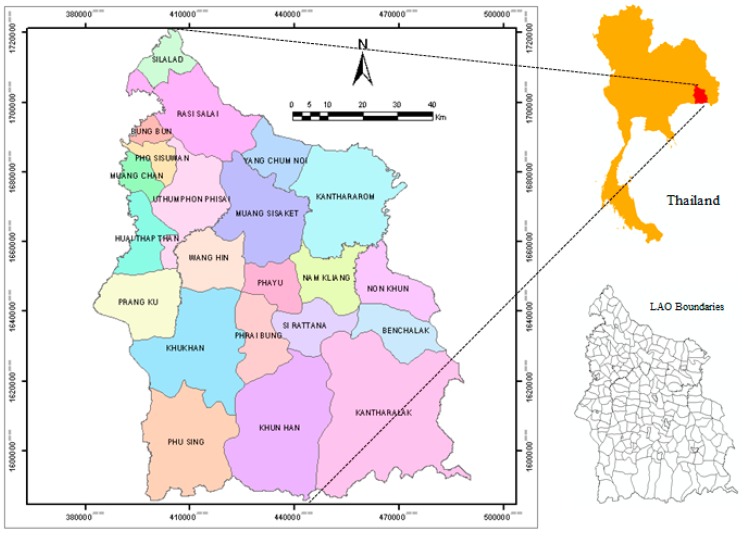
Map of Si Sa Ket province.

Si Sa Ket is located in the lower northeastern part of Thailand at the geographic location around 15°17′12″ North, 104°19′18″ East, covering the area of 8840 km² (Figure 1). It is 120 meters above mean sea level. The climate of Si Sa Ket is dry and very hot in the summer, but windy and very cold in the winter due to the influence of the northern wind from China [14]. The province is geographically divided into two parts, the north and the south, by the Moon River which flows through Rasi Salai, Mueang Si Sa Ket, Kanthararom, and Yang Chum Noi districts. The northern part of the province is covered by grassy fields, while the southern part comprises mountainous forests in Kantharalak, Khun Han and Phu Sing districts with the Phanom Dong Rak Mountain Range being a 127-kilometer borderline between Thailand and Cambodia. The topography is declined from the south toward the northern part of the province. 

There were 1,441,412 inhabitants in Si Sa Ket in 2008 [15]. It was ranked ninth largest in the country. The population density was 163 persons/km²; it was ranked the nineteenth. In terms of administration, Si Sa Ket is divided into 22 districts, which are further subdivided into 216 LAOs. There are 37 municipalities and 179 sub-district authority organizations (SAOs) altogether. Municipalities are considered more affluent areas with better accessibility to public services compared the latter ones. 

### 2.2. Data Collection and Manipulation 

All the data used in this study was secondary data collected from several organizations including TB registration data, demographic data, and spatial data. The details of data collection and management are illustrated as follows. 

#### 2.2.1. TB Registration Data

The TB occurrence data was acquired from TB clinics at district hospitals in Si Sa Ket. All the cases were collected from the Si Sa Ket TB registry, which included all confirmed TB cases notified in the residents from fiscal years 2004 to 2008. The data included every type of TB: pulmonary TB, meningitis TB, and TB in other organs. The latter two types of TB were commonly called extra-pulmonary TB (EPTB). The patients were further categorized based on their history of treatment according to the WHO and the Thailand National Tuberculosis Program (NTP) standards: new, relapsed, treatment after failure, treatment after defaulted, transferred in, and other previously treated [2,16]. To avoid duplication, only the new and relapsed cases were considered incidences in this study.

By using the standard definition of the NTP, each case record included the patient’s gender, age at diagnosis, residential address at LAO level at the time of diagnosis, and the year of diagnosis. Age was included as a covariate and was categorized according to the NTP into seven groups: (i) 0–14; (ii) 15–24; (iii) 25–34; (iv) 35–44; (v) 45–54, (vi) 55–64; and (vii) ≥ 65 years old [16].

The residential address at the time of diagnosis for each case was reviewed and assigned an LAO code number. These cases were then geo-coded and matched to the LAO-level layers of polygon by LAO code using the software ArcGIS 9.3 (ESRI Inc., Redland, CA, USA). The latitude and longitude coordinates corresponding to the centroid of each LAO were also calculated.

Personal identification of all TB cases was removed by the TB registrars, thus, no specific individual identifiers were used in the analysis. Ethical approval was, therefore, not required for this study. 

#### 2.2.2. Spatial Data

The geographic coordinates of province boundaries were collected from the Land Development Department, Ministry of Agriculture and Cooperatives. The LAOs boundary data was provided by the Department of Environment Quality Promotion, Ministry of Natural Resources and Environment, Thailand. 

#### 2.2.3. Demographic Data

The annual LAO population data was collected from the Department of Provincial Administration (DOPA), Ministry of Interior. The data was stratified into seven groups in accordance with the TB registration data. 

### 2.3. Incidence Rates Calculation and Adjustment 

It is necessary to take age into account when comparing disease incidences among different areas. If the disease is age related, a high incidence rate in a certain population might be influenced by the higher number of elderly in that population compared to others. In this respect, age standardization of the incidence rate is applied to abolish the effect. Age-standardization technique can be done in both direct and indirect manners over a selected standard population, which is used as a common reference for comparison [17].

In the indirect standardization approach, the ratio of observed to expected events was calculated, leading to the standardized morbidity ratio (SMR). To calculate the expected events, the number of the population in each age group was multiplied by the standard age specific rate yielding the case number that would be expected in that age group in the observed population [18]. In this research, an indirect standardization method was applied *vs.* direct standardization to reduce the influence of small-number effects, which could lead to an unstable rate and large errors of estimation [17,19,20]. The TB incidence cases were classified by age group to identify the overall extent of the disease without reference to the population at risk. Then, the counts of disease occurrence are converted to disease rates to compare the magnitude of the incidence among groups of population. After that, the age standardization of the TB incidence rate was applied. 

In this study, the annual mid-year population calculated from population statistics provided by the DOPA was utilized as the standard population. The SMR was calculated by using the following equation:

(1) $$Standardized\;morbidity\;ratio\left(SMR\right)=\frac{N}{\sum R_{si}P_{i}}$$

N = total number of cases in the observed population

R_si_ = age-specific incidence rate in age interval *i* in the standard population

P_i_ = the population of age interval *i* in the observed population

Assuming a Poisson distribution, the significance of the SMRs and their confidence intervals were obtained by the calculation suggested by Breslow and Day [21,22,23].

### 2.4. Spatial Pattern Detection

The detection of TB spatial patterns was divided into two parts: global detection and local detection of SMRs spatial patterns. These were accomplished using ArcGIS 9.3 cooperated with two freely distributed software packages—OpenGeoDa 1.0.1, and SaTScan v9.0.1.

#### 2.4.1. Global Detection

Global detection technique was used to examine the global pattern of TB occurrence. Spatial autocorrelation was used to assess the degree of similarity observed among a certain location and its neighboring units [17]. Moran’s I coefficient was used as the indicator in this aspect. A weight matrix was used to specify spatial relationships of the study units so that those that were close in space were given more weight in the calculation than those that were apart [24]. The queen contiguity was used because TB infection is not directional restricted [17] and the LAOs considered in this study were highly irregular in both shape and size [25]. 

Moran’s I is an extension of Pearson’s correlation coefficient to spatial neighbors. It gives a score ranging between −1 and 1. A score of zero indicates the null hypothesis of no clustering. A positive score indicates clustering of areas of similar attribute values, whereas a negative value indicates that neighboring areas tend to have dissimilar attribute values [17]. The significance of Moran’s I was assessed using Monte Carlo randomization. Computation of statistical significance assesses the significance of the Moran’s I statistic against the null hypothesis [17,24]. 

#### 2.4.2. Local Detection

In terms of spatial pattern detection of a disease, cluster analyses are important in epidemiology in order to detect aggregation of disease cases, to test the occurrence of any statistically significant clusters, and ultimately, to find evidence of etiologic factors. Cluster analysis identifies whether geographical aggregation of disease cases can be explained by chance or be statistically significant. In general, there are two types of spatial clustering methods, global and local ones. The global method is used to identify the presence of spatial clustering in the whole study area but does not specify location of the clusters. This limitation is overcome by using the local method which can pinpoint the characteristics of clusters in terms of their location, size, and magnitude [26]. 

Although the strength of Moran’s I coefficient lies in its simplicity, its principal drawback is the tendency to average local variations in situations of spatial autocorrelation. In this study, the Local Indicators of Spatial Association (LISA), which is considered the local equivalent of Moran’s I in identifying LAOs with high and low scores as well as spatial outliers, is utilized. Groups of LAOs with high scores are usually called hot spots, while the ones with low scores are cold spots, and a spatial outlier represents the location where there is a mixture of high and low scores in neighboring areas. Computation of LISA assesses the local version of Moran’s I for each location to see variation of spatial autocorrelation over the study area, meanwhile, its significance is also evaluated resulting in five scenarios of the results: High-High, Low-Low, Low-High, High-Low, and Not Significant. 

### 2.5. Spatial Scan Statistic

The spatial scan statistic was developed by Martin Kulldorff and implemented in a software program named SaTScan. SaTScan v9.0.1 was used to investigate the presence of statistically significant spatial clusters of TB and to identify their approximate locations [27]. Spatial scan statistics analyses [28] with age adjustment were performed to eliminate the influence of difference in age structure of the population. Identification of spatial high or low clusters was done under Poisson probability model assumption where the number of events in an area was Poisson distributed according to a known underlying population at risk. Purely spatial analysis, which ignored the time dimension of the cases, was performed to detect the TB clusters in the area under study. The spatial scan statistic imposed a circular window on the map and lets the circle move over the area. Therefore, at different locations, the window included different sets of neighboring LAOs. If the window contained the centroid of the LAOs, then that LAO was included in the window. The radius of each circle was increased continuously from 0 up to a maximum radius. Therefore, the window never encompassed more than 50% of the total population at risk. The likelihood function was maximized over all window positions and sizes, and the one with the maximum likelihood constituted the most likely cluster. Its significance was obtained through Monte Carlo hypothesis testing technique. To find the distribution of the test statistic, 999 random Monte Carlo replicates of the data set under the null hypothesis of no significant clusters were generated assuming that the relative risk (RR) of TB was the same within the window compared to the outside. The test statistic was calculated for each replica. In this research, the maximum size of the spatial window was set to 5% of the population because the analysis at a smaller threshold could identify smaller and more defined areas [5,29]. 

After the scanning process was accomplished, SaTScan generated the output files which included the list of LAOs and the SMRs along with their significant values. These files were then imported to ArcGIS 9.3 for visualization of the cartographic outputs. 

## 3. Results and Discussion

### 3.1. Descriptive Analysis of TB Case Notification 

**Figure 2:**
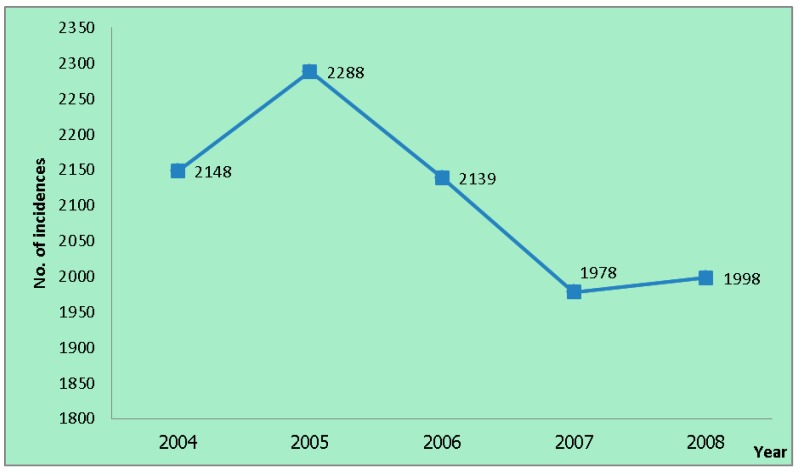
Number of tuberculosis (TB) incidences from 2004 to 2008.

There were 10,551 TB incidence cases reported in the Si Sa Ket TB clinics from 2004 to 2008. The incidence reported in 2004 was 2148 cases. It surged to the peak at 2288 cases in 2005. After that, the incidences decreased for two consecutive years at 2139 and 1978 cases, respectively. In the last year of the study period, the number of the incidences increased to 1998 cases (Figure 2). 

**Figure 3:**
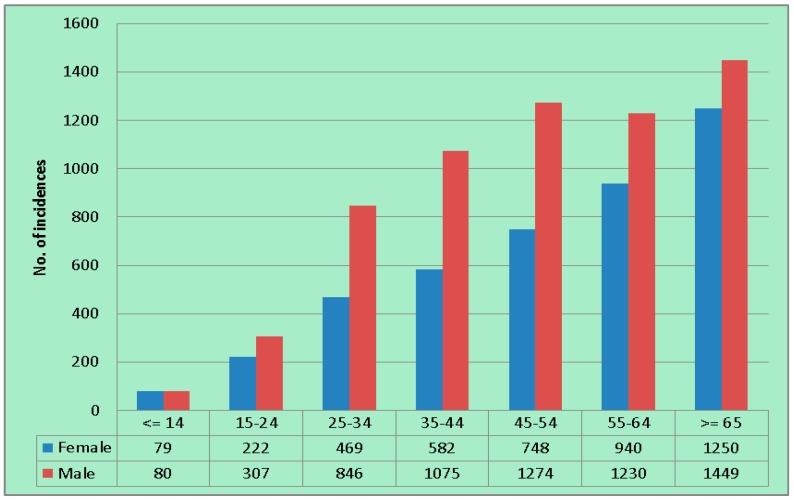
Number of total incidences by age group and gender during 2004–2008.

In terms of age and gender of the patients, the number of registered TB cases was higher in elderly groups. There were more males than female patients in every age group (Figure 3). Among the registered incidences throughout the study period, 4290 were females and 6261 were males. Generally, the proportion between females and males was around 40% to 60%. 

**Figure 4:**
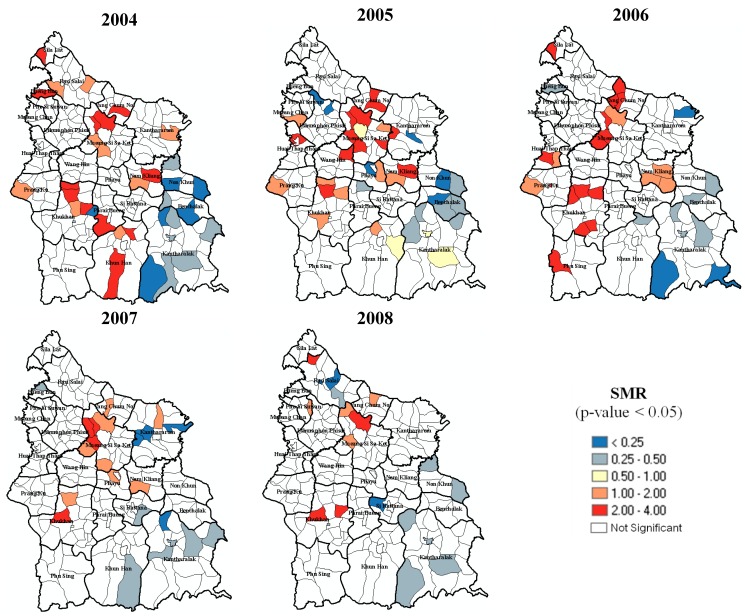
Standardized Morbidity Ratios (SMRs) of TB in Si Sa Ket from 2004 to 2008.

The number of annually registered incidence cases at the LAO level ranged from 0–62 in 2004, 0–54 in 2005, 1–51 in 2006, 0–59 in 2007, and 1–45 cases in 2008. Considering SMR of each LAOs, it ranged from 0–3.98 in 2004, 0–3.92 in 2005, 0.10–3.09 in 2006, 0–3.23 in 2007, and 0.14–3.21 in 2008. The analysis revealed that the LAOs with significantly high SMR were persistently found in the area of Mueang Si Sa Ket and Khukhan districts, while the LAOs with low SMR were mainly located around Kantharalak, Benchalak, and Non Khun districts. An overview of the SMRs (*p*-value < 0.05) is depicted in Figure 4. 

### 3.2. Spatial Pattern of TB 

After investigating TB SMRs of individual LAO, the spatial distribution pattern of the SMRs in terms of their spatial relationships—both global and local perspectives—was assessed. The results are shown in Section 3.2.1 and Section 3.2.2. 

#### 3.2.1. Global Spatial Pattern

This was the first step of the analyses—aiming to initially detect if a TB clustering pattern should be further analyzed. It was found that the values of Moran’s indices were positive (*p*-value < 0.05) in every year during the study period indicating clustered characteristic of the SMRs. Annual values of the Moran’s indices are presented in Table 1.

**Table 1:** Moran’s indices of TB incidences in Si Sa Ket, 2004–2008.

Year	Moran’s Index	Interpretation
2004	0.1888	Clustered
2005	0.2721	Clustered
2006	0.3081	Clustered
2007	0.2682	Clustered
2008	0.1910	Clustered

#### 3.2.2. Local Spatial Pattern

**Figure 5:**
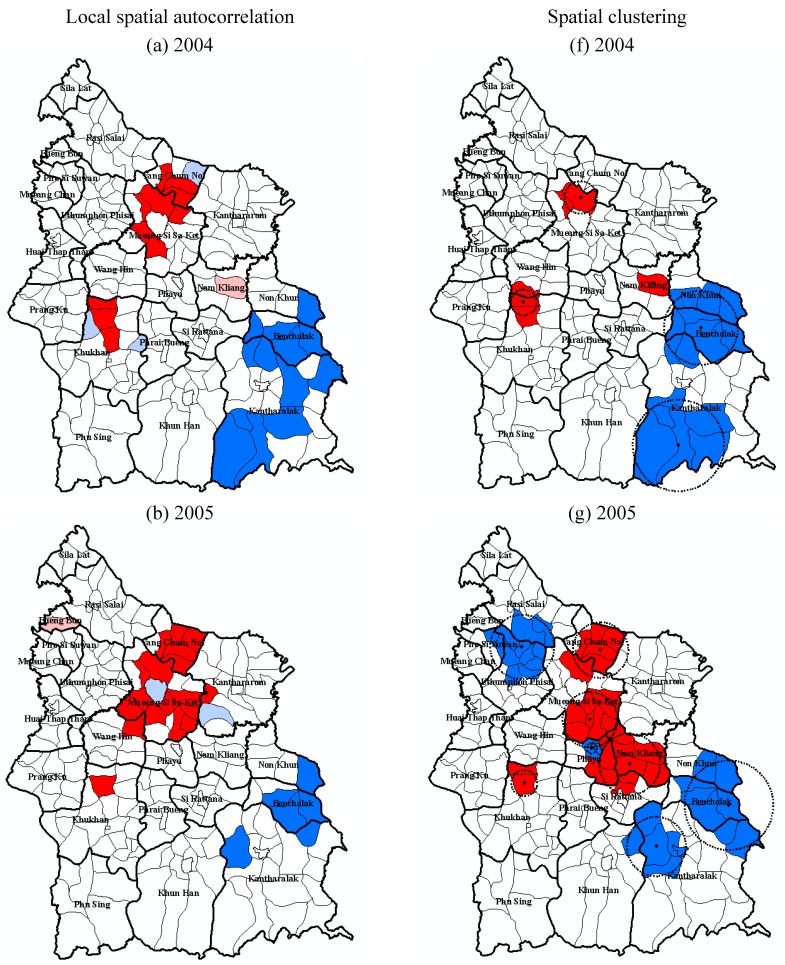

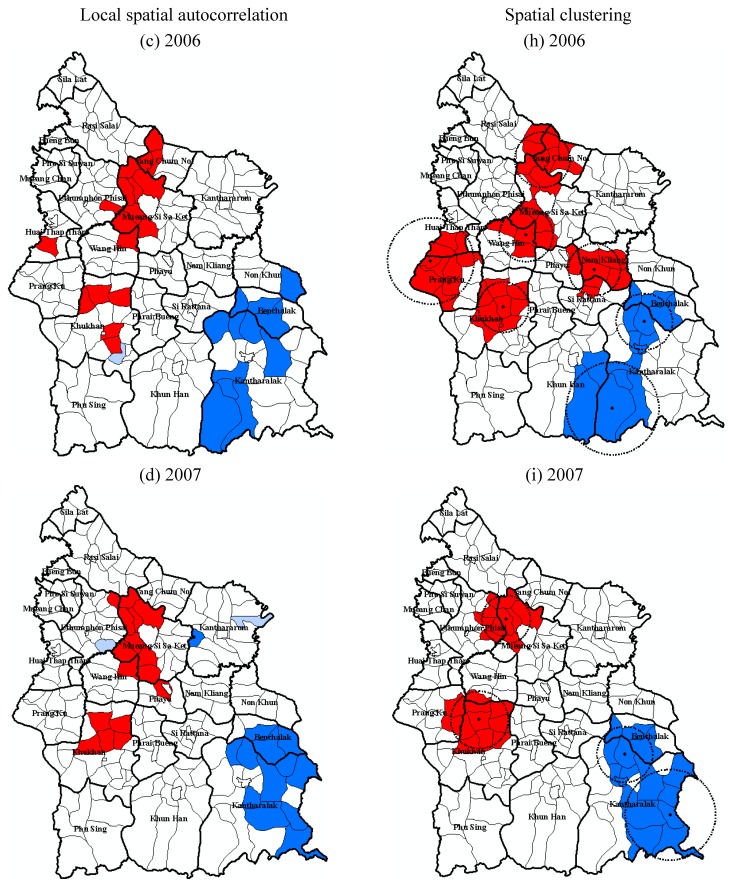

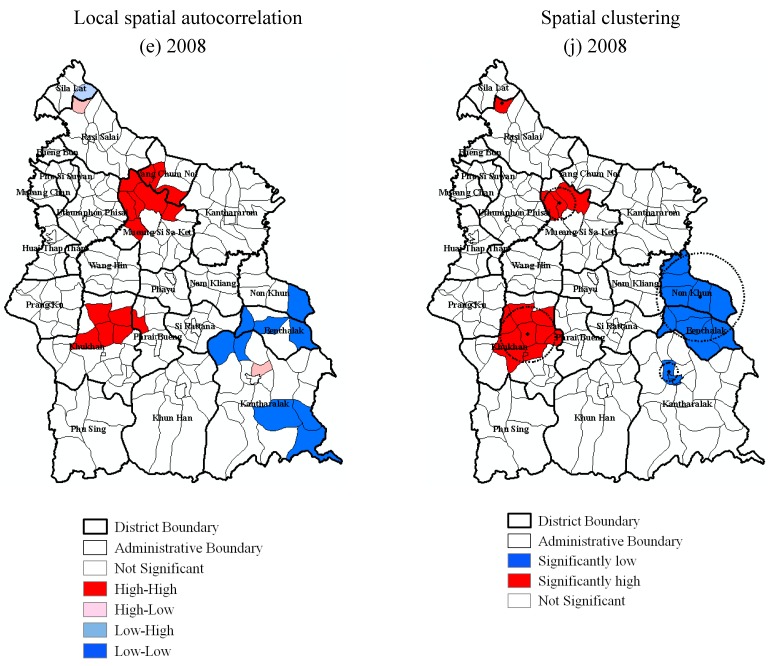
Local spatial pattern of TB incidence in Si Sa Ket from 2004 to 2008.

By using LISA, hot spots of TB incidence during 2004 to 2008 were detected in Mueang Si Sa Ket, Nam Kliang district, Khukhan, Prang Ku and Wang Hin districts, while the cold spots were mostly detected in Kantharalak, Benchalak, and Non Khun districts every year (Figure 5). These LAOs are highlighted in Figure 5a–e according to their spatial autocorrelation characteristics. 

In terms of spatial clustering detection using SaTScan software with maximum spatial cluster size of ≤5% of the total population, the spatial cluster analysis identified clusters of significantly high and low rates of TB in Si Sa Ket for 2004–2008. Results of ArcGIS mapping of spatial scan statistic were consistent with the ones generated from LISA and similar to the SMRs. Statistically significant high clusters were detected in Mueang Si Sa Ket and Khukhan districts in every year. Inversely, Kantharalak, Benchalak, and Non Khun districts fell into the significantly low clusters annually. Some distinctions of the investigation were found in Nam Kliang, Prang Ku, and Uthumphon Phisai districts. The LAOs in Nam Kliang were recognized as high-rate clusters in 2005 and 2006, the same results was also found in Prang Ku in 2006, while low clusters were detected in parts of Uthumphon Phisai and Rasi Salai in 2005 by using the SaTScan technique. These locations were considered statistically insignificant by LISA.

Figure 5 illustrates annual local patterns of TB detected by the two spatial pattern detection techniques. 

### 3.3. Discussion

#### 3.3.1. Age Standardization of TB Incidence Data

In epidemiological studies where population data is utilized to compare statistics on disease magnitude across population groups, age standardization of the data is commonly applied to minimize the influence of heterogeneity of age on the data set. Many studies suggested that the direct method of standardization should be used in adjustment of disease incidences [30,31,32] due to its strength in preservation of consistency between the populations being compared. However, several publications have complimented usage of the indirect standardization approach [33,34,35,36], especially in the situations where the population size is small or the incidence of cases is sparse (less than 20 cases). In this study, indirect age-standardization of the incidence was performed because the small number of incidences was witnessed in the study area. Instability might be found in the rates being generated by the direct method. Additionally, minimal fluctuation of the cases in a certain age group of the population in the small geographical unit like LAO might result in drastic changes in the directly standardized rates [34]. This is in accordance with the studies conducted in England and Wales, Hong Kong, and Thailand [37,38,39]. 

#### 3.3.2. Comparison between the Local Spatial Pattern Detection Techniques

LISA and SaTScan are the techniques which are commonly used in detecting local patterns of disease observations. They facilitate the identification of the location of clusters as well as the assessment of their statistical significance [40]. It was found that these two methods generated comparable results in detecting geographic areas of high-rate and low-rate clusters of TB in this study. However, some inconsistencies were found. The LISA statistic identified clusters in a more localized manner compared to SaTScan. This might be because of their different criteria in determining the clusters. LISA investigated the groups of neighboring LAOs with the rates which were significantly related to each other so that the areas with similar values were defined as cluster [41]. In this regard, LISA examined correlation between value at a certain location and the average value of neighboring locations. As for the SaTScan approach where larger clusters were detected, the maximum likelihood ratio of cases was calculated with relation to the underlying population in the area to identify the cluster of LAOs considered as having elevated or lower rates [42,43]. Similar findings were recognized in previous studies [29,42,44,45]. 

Because each method has its own principle in cluster detection, different geographic areas were selected by each method. The intersection of the selections designated that the two analytic techniques identified different features of the same clusters. These findings suggest that application of multiple methods is a preferred approach in identifying clusters of TB at the LAO level in Si Sa Ket. These spatial cluster detection techniques should be used complimentarily rather than individually. Additionally, since each approach has its benefits and disadvantages, no single method is considered the “gold standard” for cluster analysis [45].

#### 3.3.3. The Variations in the Spatial Patterns

Although the reasons for the varying patterns of SMRs within the study site were not examined, certain assumptions could be made. Clustering of LAOs with higher SMRs in Mueang Si Sa Ket and Khukhan districts can be attributed to the lower altitude of the areas. This inverse relationship is consistent with the studies which were conducted in Kenya, Mexico, Vietnam, Turkey and Nepal [46,47,48,49,50]. A possible explanation for this phenomenon is that the biological effect of the high oxygen tension at low altitude stimulates the multiplication of *Mycobacterium tuberculosis* in the lung, while the lower oxygen tension at high altitude hinders the growth rate of the tubercle bacilli [51]. Moreover, a case-control study found that the cases at high elevation had a reduction of mycobacterial multiplication in whole blood compared to the controls, and the antimycobacterial cellular immunity in the cases was intensified as well [52]. In addition to the geographic factors, several studies indicated that people with lower socio-economic status (SES) both financially and non-financially were more vulnerable to TB. It usually recognized that having low income, being illiterate, being unemployed, and residing in poor living conditions increased susceptibility to the disease [53,54,55]. Due to a lack of socio-economic data from the TB patient records in the data set being used, relationships between socio-economic factors and TB were unable to be assessed in the current study. Additional data collection and further extensive studies need to be undertaken in order to evaluate the associations between TB and its potential factors in Si Sa Ket. Nevertheless, the evident correlation with the age-standardized TB incidence being detected was the crucial findings since it revealed the locations where the public health intervention is needed. 

#### 3.3.4. Limitations

Our study had some limitations. Firstly, this study was conducted under the ecological design basis. It studied the population in groups rather than as individuals. It is intrinsically compromised in examining the complicate interaction between the various factors at the individual level. However, this type of analysis can utilize readily available data from other regular activities such as notification and census [38]. Secondly, the study period was rather short (*i.e.*, 5 years, from 2004 to 2008). Additional data for a longer period or more detailed chronological incidence data (monthly, weekly, or daily) is needed to evaluate the spatial and temporal trend of the TB pattern. 

## 4. Conclusions

This study made use of GIS and spatial analyses which have been applied to many epidemiological researches to analyze and more clearly display the spatial patterns of TB in Si Sa Ket, a highly affected province in Thailand. Both global and local techniques were applied with an intention to reveal spatial characteristics of the disease. These included Moran’s I method as a global detection tool, and Local Moran (LISA) and spatial scan statistic (SaTScan) as local detection tools. 

The results of the study suggest that there was heterogeneity of spatial pattern of TB within the study region. Retrospective purely spatial analysis showed persistence of the TB clusters in some geographical areas of the province annually. This result provides useful information on the prevailing epidemiological situation of tuberculosis in the study area. The findings, in terms of the presence of hot spots of TB in this province, can help the provincial health officers to intensify their remedial measures in the identified areas and to issue future strategies for more effective control of the disease.
